# Nonaqueous Synthesis of Pd/PdO-Functionalized NiFe_2_O_4_ Nanoparticles Enabled Enhancing n-Butanol Detection

**DOI:** 10.3390/nano14141188

**Published:** 2024-07-12

**Authors:** Hongyang Wu, Chen Chen

**Affiliations:** College of Instrumentation & Electrical Engineering, Key Laboratory of Geophysical Exploration Equipment, Ministry of Education of China, Jilin University, Changchun 130012, China; wuhx6521@mails.jlu.edu.cn

**Keywords:** nonaqueous, Pd/PdO, NiFe_2_O_4_, n-butanol, gas sensor

## Abstract

The efficient detection of n-butanol, which is in demand for highly sensitive materials, is essential for multiple applications. A nonaqueous method was applied to prepare NiFe_2_O_4_ nanoparticles (NPs) using benzyl alcohol as a solvent, which shows a size of 7.9 ± 1.6 nm and a large surface area of 82.23 m^2^/g. To further improve the sensing performance for n-butanol, Pd/PdO functionalization was sensitized with NiFe_2_O_4_ NPs. Gas sensing results demonstrate that the Pd/PdO-NiFe_2_O_4_ exhibits an enhanced response of 36.9 to 300 ppm n-butanol and a fast response and recovery time (18.2/17.6 s) at 260 °C. Furthermore, the Pd/PdO-NiFe_2_O_4_-based sensor possesses a good linear relationship between responses and the n-butanol concentration from 1 to 1000 ppm, and great selectivity against other volatile organic compounds (VOCs). The excellent sensing enhancement is attributed to the catalytic effects of Pd/PdO, the increase of oxygen vacancies, and the formation of heterojunction between PdO and NiFe_2_O_4_. Thus, this study offers an effective route for the synthesis of Pd/PdO-functionalized NiFe_2_O_4_ NPs to achieve n-butanol detection with excellent sensing performance.

## 1. Introduction

The air pollution of volatile organic compounds (VOCs) from industrial production, living organisms, and fuel combustion has attracted more and more concerns around the globe. As a typical VOC, n-butanol is an essential liquid that is widely used in plastics, rubbers, drugs, solvent extractant, and so on [1,2]. However, the vapor of n-butanol is flammable, toxic, and irritating, which endangers human health and environmental safety. One the one hand, continuous exposure to air with n-butanol may cause respiratory distress, headaches, dizziness, or even neurological damage and other symptoms. On the other hand, the mixture of n-butanol (1.45–11%) vapors with air is easily flammable and explosive when it is subjected to open fire [3]. Therefore, it is highly urgent to develop novel active sensing materials to effectively detect n-butanol gas with rapid response, high sensitivity, good selectivity, and a low detection limit.

Over the past few decades, increasing interest has been focused on the resistive gas sensor fabricated from p-type oxide semiconductors for n-butanol detection due to their distinctive charge carriers, multivalent characteristics, catalytic properties, and the formation of p-n heterojunctions [4]. As a particular type of semiconductor, cubic NiFe_2_O_4_ with various microstructures has been prepared to be efficient sensing materials to detect acetone [5], ammonia [6], toluene [7], ethyl acetate [8], etc. The existing Ni^3+^/Ni^2+^ and Fe^3+^/Fe^2+^ ions in NiFe_2_O_4_ could not only increase carrier concentration but also provide active sites, enabling it great potential as a sensing material [9]. Yet, the sensing performance of NiFe_2_O_4_ to n-butanol is rarely reported but highly attractive. NiFe_2_O_4_ nanostructures have been prepared by different methods for diverse applications. The nonaqueous synthesis in organic solvents without any water has been developed to synthesize extensive oxides. The organic parts in the reaction mixture could be both oxygen sources and capping agents to modulate the microstructure, particle size, and surface state [10,11,12]. Benzyl alcohol with a boiling point of 204.7 °C was used to directly obtain many metal oxides using organic metal salts. For example, metal oxides of ZnFe_2_O_4_ [13] NPs have been successfully prepared using benzyl alcohol under a certain temperature and pressure. Therefore, this gives us a clue to synthesize NiFe_2_O_4_ NPs using benzyl alcohol as a solvent and further explore its applications in n-butanol sensing.

Moreover, a variety of strategies have been adopted to improve the sensing properties of materials, such as creating a defect, doping, functionalizing noble particles, and forming heterostructures. The functionalization with noble metal is a simple and effective route to achieve a great improvement in sensing performance. In particular, palladium noble metal Pd could promote catalysis and sensitization, making it an excellent alternative for comprehensively improving gas sensing performance. Moreover, PdO is a typical p-type metal oxide semiconductor, which is generally produced by annealing metal Pd in air. The coupling of a small amount of PdO will not only involve a catalytic effect but also create heterojunctions with the major sensing materials, resulting in a significant improvement of gas sensors [14,15,16]. The Pd/PdO functionalization has been proved to largely improve the sensing properties of WO_3_ [17] and ZnO [18]. Nevertheless, the enhanced sensing performance of NiFe_2_O_4_ NPs functionalized with Pd/PdO for n-butanol detection has not been studied.

In this study, we report the synthesis of NiFe_2_O_4_ NPs by a nonaqueous method followed by subsequent thermal treatment, in which the nickel(II) acetylacetonate and iron (III) acetylacetonate without water were used as metal precursors and benzyl alcohol was a solvent and capping agent. The obtained NiFe_2_O_4_ NPs show promise for n-butanol detection, which can be greatly improved by the Pd/PdO functionalization, especially in terms of response and selectivity. In addition, the underlying mechanism of Pd/PdO-NiFe_2_O_4_ was explored in detail.

## 2. Experimental Section

### 2.1. Materials Synthesis

All chemical materials, such as iron (III) acetylacetonate, nickel(II) acetylacetonate, palladium chloride (PdCl_2_), benzyl alcohol, and anhydrous ethanol, were purchased form the Aladdin (Shanghai, China) and are all analytical reagent (AR) and used directly. Deionized water (18 MΩ) was used in all experiments. The NiFe_2_O_4_ NPs were synthesized by a solvothermal method and subsequent heat treatment. Specifically, 0.5 mmol nickel(II) acetylacetonate and 1 mmol iron (III) acetylacetonate were completely dissolved into 50 mL benzyl alcohol with vigorous stirring for 1 h. Then, the solution was transferred into 100 mL Teflon-lined stainless-steel autoclave and incubated at 200 °C for 24 h. After the autoclave cooled down to room temperature, the products were centrifugally collected by washing several times with acetone, deionized water, and ethanol. Finally, the obtained product was dried in an oven at 60 °C and then annealed in air at 400 °C for 2 h at a heating rate of 2.0 °C/min to obtain NiFe_2_O_4_. Pd/PdO-NiFe_2_O_4_ sample was synthesized by following the identical process except for the addition of 0.1 mmol PdCl_2_.

### 2.2. Material Characterization

A variety of analytical methods have been used to characterize the as-synthesized samples. The crystallographic phases of the samples were tested by X-ray diffraction (XRD, D8-Advance, Cu-Kα, λ = 0.1542 nm), and a scanning rate of 1°/min was set. The microstructures were observed by a transmission electron microscopy (TEM) and high-resolution TEM (HRTEM) on a JEM-2010HR apparatus. The specific surface area and pore distribution were measured by a nitrogen adsorption/desorption measurement at 77.3 K via an ASAP 2020 sorption system. Thermogravimetric analysis (TGA) was carried out in a stream of air from room temperature to 600 °C with a heating rate of 10 °C/min. The electron paramagnetic resonance (EPR) spectra were tested by a Bruker A300 spectrometer. The composition and chemical states of the elements on the surface of the sample were analyzed by X-ray photoelectron spectroscopy (XPS, Thermo Kalpha, Al Kα = 1486.6 eV). All binding energies were calibrated related to characteristic C 1s peak at 284.6 eV.

### 2.3. Fabrication and Measurement of Gas Sensor

The gas sensor was fabricated by the following steps. First, a dense slurry was formed by fully mixing a suitable amount of obtained powder with ethanol. Second, the slurry was cast onto the surface of an alumina ceramic tube with two gold electrodes by a brush. The sensor was first dried at 100 °C for 2 h and then annealed at 400 °C for another 2 h before gas sensing measurement. A Ni-Cr heating wire inserted in the tube was used to control the operating temperature of the sensor by varying the current. We measured the resistance of gas sensors with a WS-30 A system (Weisheng Instruments Co., Zhengzhou, China). During the test, a desired concentration was obtained by evaporating a specific quantity of liquid volatile organic compounds (VOCs) to mix with dry air, which was calculated by the following equation [19]:

(1) $$C\left(ppm\right)=\frac{V_{x}\left(mL\right)\times P\times \rho \left(g/mL\right)\times 22.4\left(L/mol\right)}{M\left(g/mol\right)\times V\left(L\right)}\times 10^{6}\left(ppm\right)$$

where C (ppm) is the gas concentration, V (L) is the total volume of the chamber, and V_x_ (mL), P, ρ (g/mL), and M (g/mol) are the volume, purity, density, and molecular weight of the liquid, respectively.

The sensitive response of the sensor was calculated by the alteration in resistance. For a p-type semiconductor in a reducing atmosphere, the response can be expressed as:

(2) $$S=\frac{R_{g}}{R_{a}}$$

where R_a_ and R_g_ are the resistance values of the sensor in air and analytic gas, respectively. In addition, the response time (*τ_res_*) and recovery time (*τ_rec_*) refer to the time required for the sensor to achieve 90% of the resistance change during the response and recovery processes. During the measurements, the relative humidity was maintained to be around 30%.

## 3. Results and Discussion

### 3.1. Structural and Morphological Characterization

**Figure 1:**
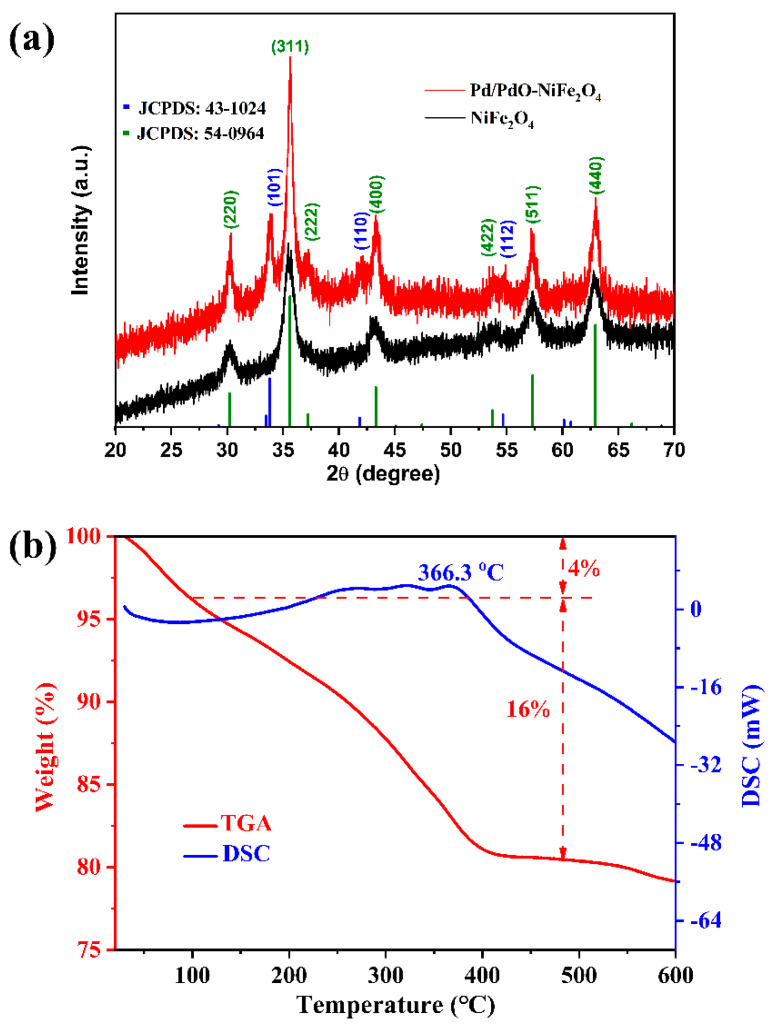
(**a**) XRD patterns of the NiFe_2_O_4_ and Pd/PdO-NiFe_2_O_4_ NPs, and (**b**) thermogravimetric analysis of the formation of NiFe_2_O_4_.

The as-prepared NiFe_2_O_4_ and Pd/PdO-NiFe_2_O_4_ NPs were first examined by XRD to study the crystalline phase, as shown in Figure 1a. The diffraction peaks at 30.3°, 35.6°, 37.2°, 43.4°, 53.7°, 57.4°, and 63.0° are originated from the reflection of (220), (311), (222), (400), (422), (511), and (440) planes of cubic NiFe_2_O_4_ (JCPDS: 54-0964). Regarding Pd/PdO-NiFe_2_O_4_, additional peaks located at 33.8°, 42.2°, and 54.8° are detected, which are assigned to the (101), (110), and (112) crystal planes of PdO (JCPDS: 43-1024), respectively. However, there is no diffraction peak of metal Pd, which may be attributed to the lower amount and the coverage of the PdO layer. These XRD patterns clearly indicate the successful synthesis of NiFe_2_O_4_ and Pd/PdO-NiFe_2_O_4_ by the nonaqueous route in benzyl alcohol followed by thermal treatment.

The thermogravimetric analysis for the crystallization of NiFe_2_O_4_ is shown in Figure 1b. A slight 4% weight loss is observed below 150 °C because of the evaporation of residual water in the dried precursor. Then, a major weight loss of about 16% occurs when the temperature is increased to around 480 °C, due to the decomposition of organic parts in the precursor and the formation of crystal NiFe_2_O_4_. Meanwhile, from the DSC curve, an exothermic peak is detected at 366.3 °C. Many simple oxides can be directly prepared in the organic solvent, such as benzyl alcohol [10,11,12], at a lower temperature; however, subsequent thermal treatment is required for the crystallization of NiFe_2_O_4_ due to the higher reaction temperature of NiFe_2_O_4_ NPs by a nonaqueous solvothermal method [20].

**Figure 2:**
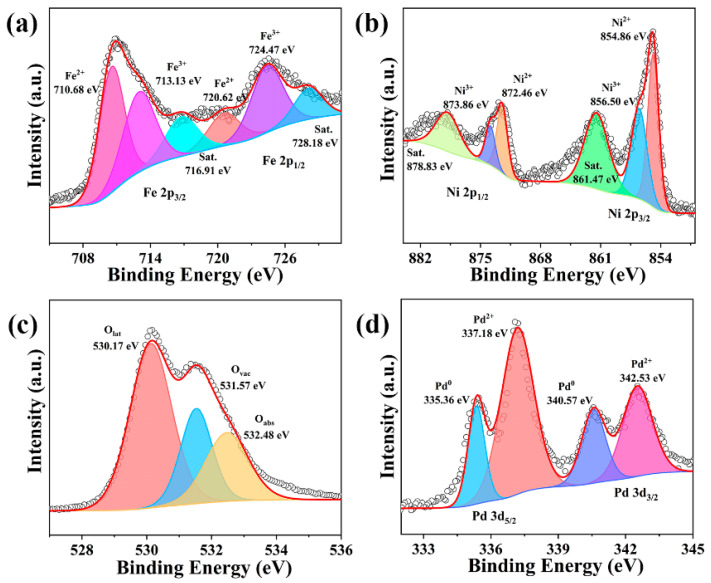
XPS spectrum of (**a**) Fe 2p, (**b**) Ni 2p, (**c**) O 1s, and (**d**) the Pd spectra for Pd/PdO-NiFe_2_O_4_ NPs, respectively.

**Figure 3:**
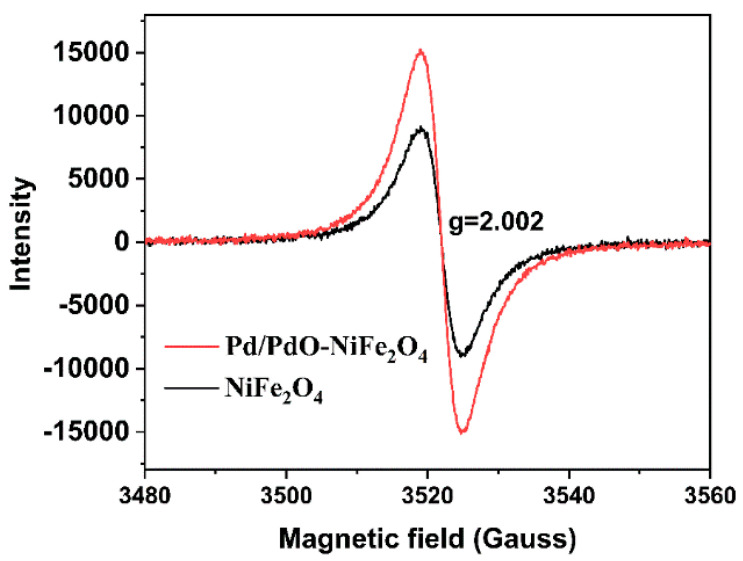
EPR spectra of NiFe_2_O_4_ and Pd/PdO-NiFe_2_O_4_ NPs.

XPS analysis was performed to check the composition and valence states of Pd/PdO-NiFe_2_O_4_ NPs. Figure 2a shows that the fitting peaks of Fe 2p at 710.68 and 720.62 eV are characteristic of Fe^2+^, and other peaks at 716.91 and 728.18 eV are satellite peaks. The peaks located at 713.13 and 724.47 eV are identical to Fe^3+^ [21]. Figure 2b shows the high resolution of the Ni 2p spectrum. The peaks at 854.86 and 872.46 eV are ascribed to Ni^2+^ accompanied by a pair of satellite peaks at 861.47 and 878.83 eV. The binding energies centered at 856.50 and 873.86 eV can be assigned to Ni^3+^. In Figure 2c, the O 1s spectrum is decomposed into two peaks. The lattice oxygen (O_lat_) is located at 530.17 eV, which is unreactive to affect the conductivity of the sensing materials. In contrast, the chemisorbed oxygen with a higher binding energy of 532.48 eV will evolve into active oxygen species of O_2_^−^, O^−^, and O^2−^, which could be key factors to determine the gas sensing performance [22]. Notably, the peak related to oxygen vacancies (Ov) is observed at 531.57 eV. Figure 2d shows the spectrum of Pd 3d, in which 3d_5/2_ and 3d_3/2_ at binding energies of 337.18 and 342.53 eV verify the Pd^2+^ state [23,24]. The second pair of peaks, with a low binding energy of 335.36 and 340.57 eV, is associated with the Pd^0^ state, suggesting the existence of considerable metal Pd. Because of the reductive hydroxyl of benzyl alcohol, the Pd nanoparticles could be obtained and were further oxidated into PdO during the thermal treatment in air at 400 °C. However, the formation of an outer PdO layer may prevent its complete conversion, resulting in the formation of Pd/PdO. The co-existence of Pd and PdO is beneficial for the generation of oxygen species and further promotes their reactions with target gas molecules. The abundant oxygen species, the catalytic sensitization of Pd/PdO, and the reversible redox reaction between Ni^2+^ and Ni^3+^, and Fe^2+^ and Fe^3+^ could be favorable for the reaction of n-butanol molecules on the surface of NiFe_2_O_4_, thus improving the sensing properties [25]. EPR spectroscopic measurements were recorded to confirm the oxygen vacancies in NiFe_2_O_4_ and Pd/PdO-NiFe_2_O_4_ NPs. In Figure 3, a clear EPR signal is observed with a g factor of 2.002, which is derived from unpaired electrons in the oxygen vacancy sites [26]. It is seen that there are oxygen vacancies in the NiFe_2_O_4_ and the intensity is increased from 9056 to 15,219, indicating the increasement in oxygen vacancies by Pd/PdO functionalization, which is greatly beneficial for enhancing the sensing performance.

**Figure 4:**
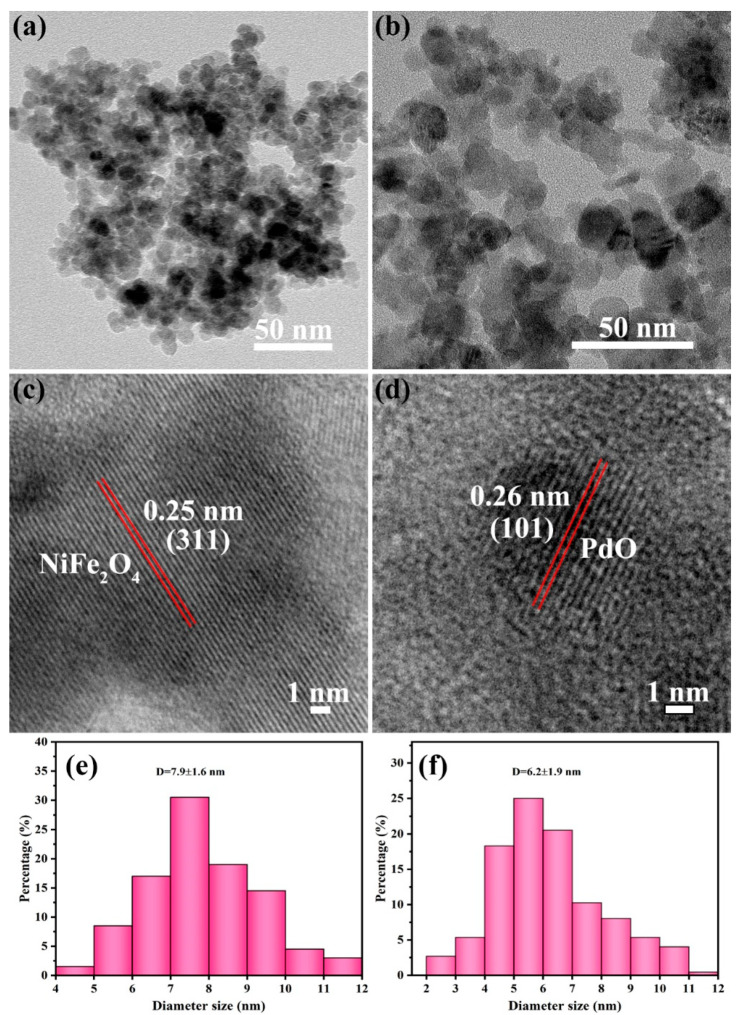
The TEM images and size distribution of (**a**) NiFe_2_O_4_ and (**b**) Pd/PdO-NiFe_2_O_4_ NPs, the HRTEM images of (**c**) NiFe_2_O_4_ and (**d**) PdO, and the size distribution of (**e**) NiFe_2_O_4_ and (**f**) Pd/PdO-NiFe_2_O_4_.

**Figure 5:**
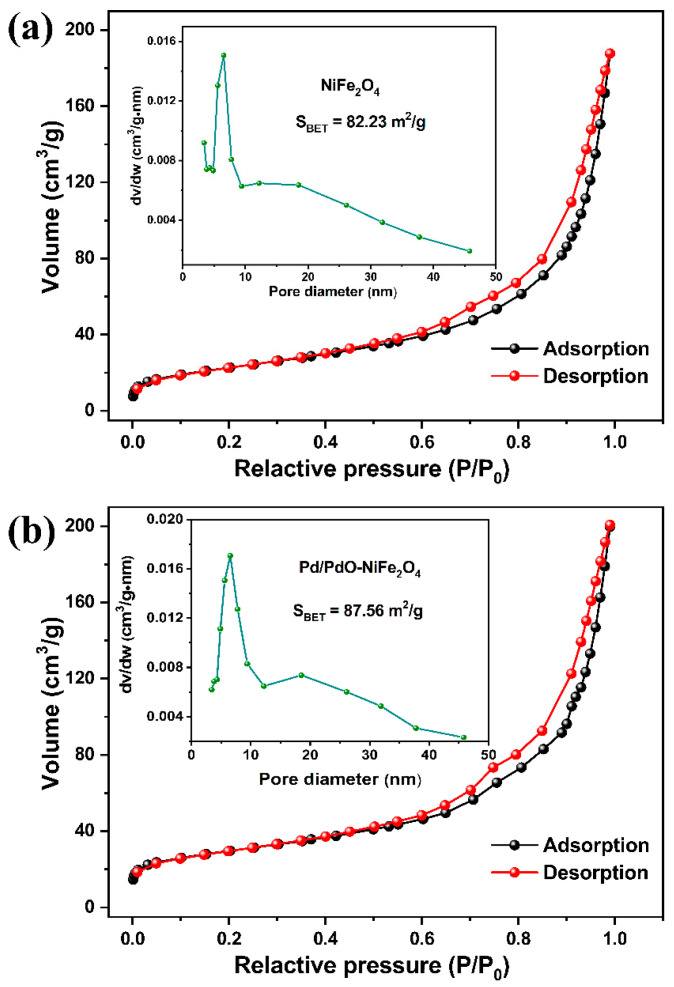
Typical nitrogen adsorption/desorption isotherms, and the inset displays the pore size distribution plot of (**a**) NiFe_2_O_4_ and (**b**) Pd/PdO-NiFe_2_O_4_ NPs.

TEM images in Figure 4a,b show the NPs of NiFe_2_O_4_ and Pd/PdO-NiFe_2_O_4_, and their average diameters are found to be around 7.9 ± 1.6 (Figure 4e) and 6.2 ± 1.9 nm (Figure 4f), respectively. It is worthwhile to note that the average size is slightly reduced after the introduction of PdO. The HRTEM image of NiFe_2_O_4_ NPs exhibits adjacent lattice fringes with an interplanar distance of 0.25 nm (Figure 4c), corresponding to the (311) facet of NiFe_2_O_4_. The lattice spacing of 0.26 nm can also be found for the (101) plane of PdO (Figure 4d). This analysis is consistent with XRD results, which again verify the synthesis of NiFe_2_O_4_ and Pd/PdO-NiFe_2_O_4_ with high crystallization. Furthermore, the nitrogen adsorption/desorption measurement was used to analyze the specific surface area and the pore size. The BET surface area of NiFe_2_O_4_ and Pd/PdO-NiFe_2_O_4_ NPs is determined to be 82.23 and 87.56 m^2^/g, and the pore size distribution is in the range of 4–9 nm (Figure 5). The large accessible surface area and mesoporous structures could provide a large number of active sites for gas interaction and facilitate the gas diffusion, contributing to the enhanced sensing properties.

### 3.2. Gas Sensing Performance

**Figure 6:**
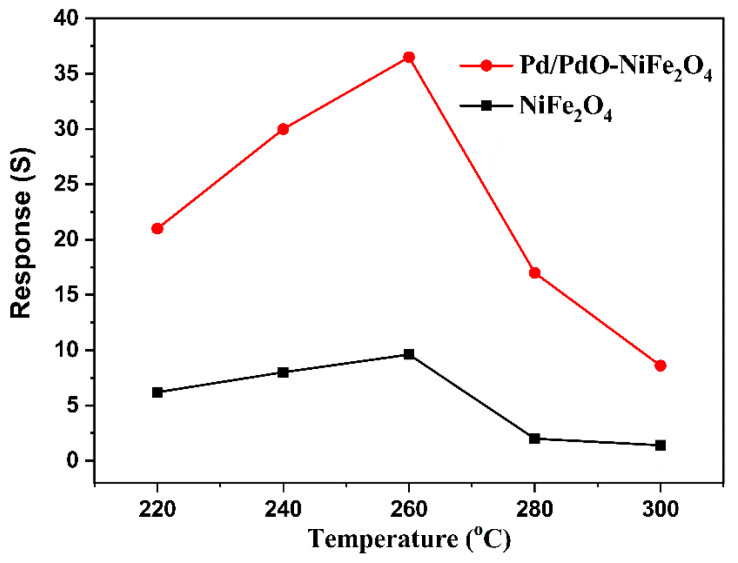
The response of the sensors based on NiFe_2_O_4_ and Pd/PdO-NiFe_2_O_4_ NPs to 300 ppm n-butanol between 220 and 300 °C.

The responses of resistive gas sensors are heavily dependent on operating temperature, which affects the reaction of gas molecules on the surface of sensing materials. Thus, we have examined the effects of operating temperature on sensors based on NiFe_2_O_4_ and Pd/PdO-NiFe_2_O_4_ with 300 ppm n-butanol, and the results are plotted in Figure 6. The responses of two sensors increase with rising temperature and then reach their optimal operating temperature. The lower operating temperature is not conducive to promoting the reaction and the adsorption/desorption of gas molecules, leading to a poor sensing performance. The maximum responses of NiFe_2_O_4_ and Pd/PdO-NiFe_2_O_4_ are 9.6 and 36.9 at 260 °C, respectively, in which the Pd/PdO-NiFe_2_O_4_ shows enhanced sensing performance. With the further increase in temperature, the responses decrease gradually due to the fast desorption of gas molecules.

**Figure 7:**
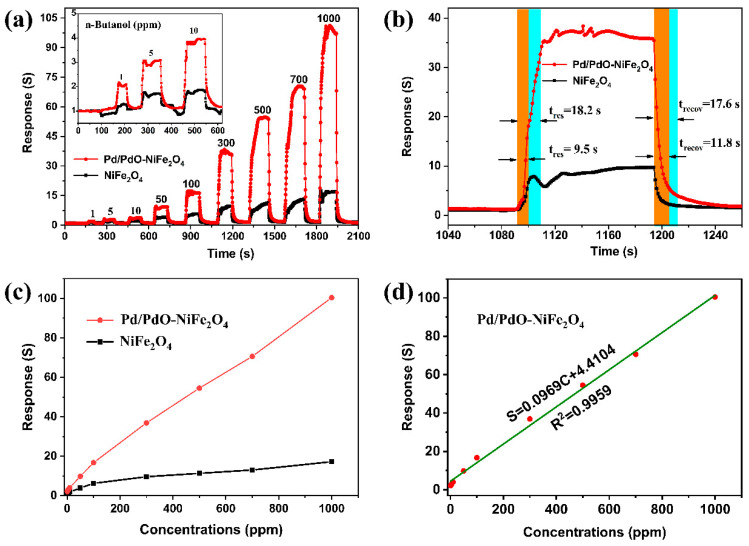
Dynamic response curves (**a**), response time and recovery time (to 300 ppm n-butanol), (**b**) the relationship between the responses and the n-butanol concentrations (**c**) of the sensors based on NiFe_2_O_4_ and Pd/PdO-NiFe_2_O_4_ toward n-butanol, and the linear fitting of Pd/PdO-NiFe_2_O_4_ response to various n-butanol concentrations (**d**) at optimal operating temperatures of 260 °C.

**Figure 8:**
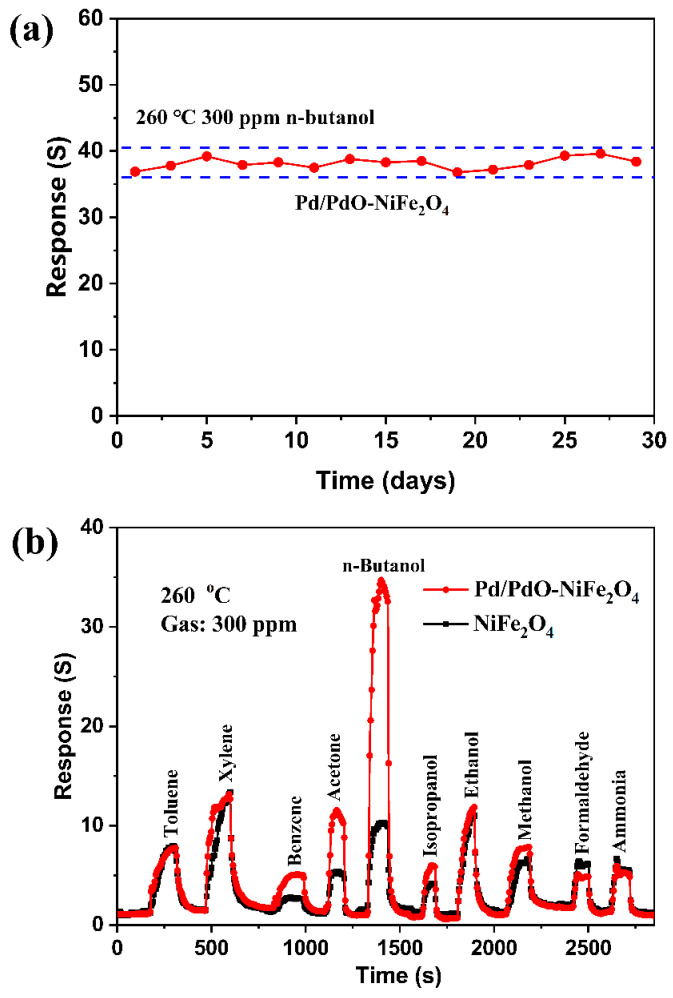
(**a**) Long-term stability of Pd/PdO-NiFe_2_O_4_-based gas sensor, and (**b**) responses to 300 ppm different VOCs gases of NiFe_2_O_4_ and Pd/PdO-NiFe_2_O_4_ at 260 °C.

The dynamic response/recovery curves of two sensors for various n-butanol concentrations in the range of 1 to 1000 ppm at 260 °C are shown in Figure 7a. Obviously, their response increases with increasing gas concentration and do not reach a saturation. The Pd/PdO-NiFe_2_O_4_ exhibits a higher response in each concentration of n-butanol compared to NiFe_2_O_4_. The relationship between the responses and different n-butanol concentrations is plotted in Figure 7c. The responses of the Pd/PdO-NiFe_2_O_4_ sensor at 1–1000 ppm n-butanol are 2.1, 3.2, 4.0, 9.8, 16.7, 36.9, 54.5, 70.6, and 100.4, respectively, indicating the high response to different n-butanol concentrations. Moreover, the response/recovery times of NiFe_2_O_4_ and Pd/PdO-NiFe_2_O_4_ toward 300 ppm n-butanol are 9.5/11.8 and 18.2/17.6 s (Figure 7b), respectively. Especially, the Pd/PdO-NiFe_2_O_4_ shows a response value of 2.1 to 1 ppm n-butanol, which is higher than that of 1.26 of pure NiFe_2_O_4_ NPs, demonstrating the low limit of detection (LOD) capability for n-butanol detection. The relationship between the responses (S) and the n-butanol concentrations (C) from 1 to 1000 ppm can be linearly fitted (R^2^ = 0.9959) for the Pd/PdO-NiFe_2_O_4_ sensor (Figure 7d) as follows:

S = 0.09669C + 4.4104(3)

which suggests the relatively rapid adsorption rate of oxygen [8]. Figure 8a displays the long-term stability of the Pd/PdO-NiFe_2_O_4_ sensor upon exposing to 300 ppm n-butanol for 30 days. Only a slight fluctuation in response values is observed, demonstrating a good long-term stability for practical applications. A comparison of sensing performances between our sensor and other p-type semiconductors such as Co_3_O_4_, NiO, CuO, LaFeO_3_ and their compositions to n-butanol detection is listed in Table 1. It is clearly seen that the sensor based on Pd/PdO-NiFe_2_O_4_ exhibits a comparably excellent performance, especially in terms of response and detection limit, holding great potential for practical application.

Selectivity is another important criterion to assess the sensing performance as well. To evaluate the selectivity of NiFe_2_O_4_ and Pd/PdO-NiFe_2_O_4_, the responses to 300 ppm of various VOCs gases are performed, as shown in Figure 8b. The sensor based on Pd/PdO-NiFe_2_O_4_ shows a much higher response of 36.9 than to toluene (7.9), xylene (13.3), benzene (5.4), acetone (11.6), isopropanol (6.2), ethanol (12.3), methanol (7.9), formaldehyde (5.1), and ammonia (5.3). However, the sensor based on NiFe_2_O_4_ exhibits poor selectivity. This enhanced n-butanol selectivity of Pd/PdO-NiFe_2_O_4_ is mainly attributed to the highly catalytic activity of PdO [18,36,37] to n-butanol molecules and the formation of heterojunctions between PdO and NiFe_2_O_4_.

### 3.3. Gas Sensing Mechanism

The gas sensing mechanism is widely interpreted based on the resistance change, which is caused by the adsorption/desorption and its chemical reactions of gas molecules on the surface of sensing material [38]. As NiFe_2_O_4_ is a typical p-type semiconductor, the chemisorbed oxygen molecules will evolve into oxygen species (O_2_^−^, O^−^, and O^2−^) at a certain temperature after capturing electrons from the sensing layer in air. The consumption of electrons leads to the formation of a deletion layer between PdO and NiFe_2_O_4_ and the increase in hole concentration; as a result, the sensor shows low resistance. When the sensor is exposed to reducing gas such as n-butanol, it can react with those active oxygen species, which results in the release of captured electrons and the increase in the resistance of the gas sensor. These processes can be expressed as follows [3,39]:

(4) $$O_{2\left(gas\right)}\to O_{2}\left(ads\right)\;\left(T<100\;{}^{\circ}C\right)$$

(5) $$O_{2}\left(ads\right)+e^{-}\to O_{2}^{-}\left(ads\right)\;\left(100<T<200\;{}^{\circ}C\right)$$

(6) $$O_{2}^{-}\left(ads\right)+e^{-}\to 2O^{-}\left(ads\right)\;\left(200<T<300\;{}^{\circ}C\right)$$

(7) $$O^{-}\left(ads\right)+e^{-}\to O^{2-}\left(ads\right)\;\left(T<300\;{}^{\circ}C\right)$$

(8) $$CH_{3}{\left(CH_{2}\right)}_{3}OH+12O^{2-}\left(ads\right)\to 4CO_{2}+5H_{2}O+12e^{-}$$

The large surface area and rich chemical state in NiFe_2_O_4_ NPs make it a good candidate for n-butanol detection. The enhancement in n-butanol sensing characteristics of Pd/PdO-NiFe_2_O_4_ can be ascribed to the following main reasons. First, the oxygen state is modulated by the Pd/PdO functionalization. The Pd and PdO possess superior catalytic effects in the oxidation of VOCs. As XPS and EPR analysis show, the Pd/PdO functionalization could provide specific sites and oxygen vacancies to adsorb and decompose more oxygen and n-butanol molecules. The active oxygen species are easily adsorbed on Pd and PdO, and then promote the reaction and charge transfer. Furthermore, the Pd and PdO will reduce the activation barrier for the decomposition of n-butanol gas. As a result, more effective reactions between adsorbed oxygen and n-butanol take place on the surface of sensing materials, which is eventually favorable to detecting n-butanol. Second, these oxygen vacancies are prone to trap electrons to generate more oxygen species to promote the reaction with n-butanol molecules [40]. Third, the contact of PdO and NiFe_2_O_4_ could create heterojunctions. The difference in work function of PdO (7.9 eV) [37] and NiFe_2_O_4_ (4.61 eV) [41] will drive the flow of holes and electrons to achieve an equilibrium in Fermi energy levels [42]. This process could increase the depletion layer and potential energy barrier at the interface between PdO and NiFe_2_O_4_, resulting in a big resistance change when the target gas is introduced to achieve a high response for n-butanol detection. In addition, the hetero-interface provides additional active regions for oxygen molecules and n-butanol gas adsorption, which is favorable for their reaction to improve the sensing properties [4,43]. Moreover, the transfer of the charge carriers is enhanced through the interfaces during the reaction between n-butanol molecules and the active oxygen species [43,44], enhancing sensing performance.

## 4. Conclusions

To summarize, a solvent thermal synthesis combined with subsequent annealing was performed as a facile method for the preparation of NiFe_2_O_4_ NPs, in which the Pd/PdO functionalization was easily introduced. Benefiting from the heterojunctions of PdO-NiFe_2_O_4_ and the catalytic properties of Pd/PdO, the Pd/PdO-NiFe_2_O_4_ enables an enhanced wide n-butanol detection from 1 to 1000 ppm. In particular, the oxygen vacancies are increased after Pd/PdO functionalization, which is favorable for the enhancement in sensing properties. As a result, the Pd/PdO-NiFe_2_O_4_-based sensor shows a high response value (36.9 to 300 ppm), low detection limit (2.1 to 1 ppm), fast detection (18.2/17.6 s), good linearity from 1 to 1000 ppm, and enhanced selectivity to n-butanol against interfering VOCs gases at the optimum working temperature of 260 °C. Hence, our work offers a novel strategy for the synthesis of NiFe_2_O_4_ NPs functionalized with Pd/PdO to fabricate a sensitive and selective n-butanol sensor.

## Figures and Tables

**Table 1:** Comparison of n-butanol sensing characteristics of different p-type gas sensors.

Materials	Microstructure	C (ppm)	T (°C)	Response	τ_res_/τ_rec_ (s)	LOD (ppm)	Ref.
CeO_2/_Co_3_O_4_	Micro-flower	100	350	87.96	63/11	2	[27]
Co_3_O_4_@ZnO	Hollow sphere	100	275	260	1/92	0.1	[3]
Co_3_O_4_	Nanosphere	5	100	86.1		0.4	[28]
Co_3_O_4_	Nanosphere	100	140	53.78	99/50	0.15	[29]
CuO	Micro-sheet	1000	160	69.73		10	[30]
NiFe_2_O_4_	Nanoblock	10	130	29.747	16/955		[31]
Co_3_O_4_	Micro-flower	100	165	8.43	59/63		[32]
Fe-doped NiO	Flower-like	100	275	114	63/21	0.05	[33]
Au-LaFeO_3_	Core-shell spheres	100	225	115		0.5	[34]
PtO_2_/CuO	Polyhedron	100	180	11.55	2.4/5.1		[35]
Pd/PdO-NiFe_2_O_4_	NPs	300	260	36.9	18.2/17.6	1	This work

## Data Availability

Data is contained within the article.
